# Optimization of Antibacterial Activity in Tibetan Swine α-Helix Peptide TP by Site-Directed Mutagenesis

**DOI:** 10.3389/fmicb.2022.864374

**Published:** 2022-07-04

**Authors:** Guoyu Li, Xiaojie Yuan, Hongyu Chen, Bowen Li, Changxuan Shao, Yongjie Zhu, Zhenheng Lai, Anshan Shan

**Affiliations:** Institute of Animal Nutrition, Northeast Agricultural University, Harbin, China

**Keywords:** TP, antimicrobial peptides, site-directed mutagenesis, antibacterial activity, treatment potential

## Abstract

Antimicrobial peptides (AMPs) have attracted extensive attention because of their broad-spectrum antibacterial activity and low level of induced bacterial resistance. However, the development of some natural AMPs does not consider the perfect balance of structural characteristics, resulting in some empirical and controversial practices still existing. To further explore and complete the relationship between parameters and function of α-helix peptide, in this study, the natural antimicrobial peptide TP secreted from Bacillus strain of Tibetan pigs was selected as a template to investigate the effect of systematic mutations in the hydrogen bond formation site of the α-helical antimicrobial peptide on the activity and cell selectivity of the antimicrobial peptide. The target peptide TP(i+4) 1&2&5 with modification of two pairs of positively charged amino acids and a pair of hydrophobic amino acids showed excellent antibacterial ability and the best selectivity index (SI = 64) *in vitro*. At the same time, TP(i+4) 1&2&5 remained active in the presence of physiological salts and serum. The results of fluorescence, flow cytometry, and electron microscopy showed that the optimized sequences showed good antibacterial activity by membrane infiltration and membrane destruction. The potential of TP(i+4) 1&2&5 *in vivo* was tested in a mouse peritonitis model. Organ bacterial loads in the liver, kidney, spleen, and lungs of mice treated with TP(i+4) 1&2&5 were significantly lower compared to the infected group (*p* < 0.05). Overall, these findings contribute to the design and optimization of antimicrobial peptides with high activity and low toxicity and may accelerate the clinical application of antimicrobial peptides.

## Introduction

The overuse use of antibiotics promotes the emergence of drug residues and bacterial resistance, and the increasing number of drug-resistant bacteria is seriously threatening public health (Wiradharma et al., 2011; Wang et al., 2020; Fang et al., 2021; Lai et al., 2021). Therefore, it is more imperative than ever to design and identify alternative antibacterial drugs with new bactericidal modes.

Antimicrobial peptides (AMPs) exist widely in many kinds of organisms and an important part of the human body to resist the invasion of pathogenic microorganisms (Brown and Hancock, 2006). They not only have direct activity on bacteria, fungi, and viruses, but also have indirect immunomodulatory activity in the host (Mishra et al., 2019; Mookherjee et al., 2020). AMPs typically achieve antimicrobial effects by physically disrupting microbial membranes and inducing leakage of cellular contents, so they have a lower tendency to induce bacterial resistance than traditional antibiotics (Rončević et al., 2018). At present, to develop high-efficiency AMPs with practical application potential, modification of natural α-helix cationic AMPs has become a hot research object. In fact, there are quite a number of peptides that reveal weak antibacterial activity and are active against host cells causing toxicity (Joo et al., 2016). Several studies have proven that the degrees of antibacterial potency and cell selectivity are determined by the peptide physicochemical properties such as length, charge, secondary structure, hydrophobicity, and amphipathicity (Chen et al., 2007; Ma et al., 2015). With the development of the research, the enhancement of the efficacy of antimicrobial peptides is no longer the pursuit of the maximum value of efficacy due to the change of a single factor, but requires system transformation to find the optimal balance. Amphiphilicity is an important structural parameter of spiral AMPs (Gong et al., 2019; Lai et al., 2022). Amphiphilic α-helix AMP binds to the bacterial membrane by electrostatic attraction, and then, the hydrophobic end is inserted into the plasma membrane to destroy the bacterial membrane structure (Brogden, 2005; Findlay et al., 2010; Wiradharma et al., 2013). Previous research shows that destroying the perfect affinity of antimicrobial peptide can improve the activity of antimicrobial peptide, to explore the effect of different hydrophobic amino acid substitutions for amino acids in the hydrogen bond formation position of α-helical peptides on the antimicrobial activity of incompletely amphiphilic antimicrobial peptides. The results show that Trp to improve antibacterial activity is the most obvious (Zhu et al., 2014, 2015). However, there are few studies on perfect amphiphilic peptides.

In this research, through the understanding of the folding principle of helix peptide, the peptide carbonyl O atom and amide proton between the ith and (i+4) th amino acid positions form a paired hydrogen bond (H–H). To further explore the relationship between the structure and function of α-helical antimicrobial peptides, we selected the natural α-helical peptide TP. TP (ASVVNKLTGGVAGLLK) consists of 16 amino acids and is a natural AMPs secreted by Bacillus strains in the intestinal tract of Tibetan pigs (Xin et al., 2017). The hydrophobic end and the positively charged end are distributed on both sides, respectively, presenting a perfect amphiphilic α-helix structure. To exclude the influence of different amino acids on the experiment, Lys of TP peptide itself was selected as the substituted positively charged amino acid. Based on the effect of positive charge at H-H position on polarity surface, in the first step, all paired amino acids at the hydrogen bond formation positions (i, i+4) on the polar surface were replaced with Lys, respectively. Specifically, Lys substituted pairs of amino acids at the N5 G9, G9 G13, S2 K6, and K6 G10 sites on the TP peptide and named as TP(i+4) 1, TP(i+4) 2, TP(i+4) 3, and TP(i+4) 4. Subsequently, two and three pairs of different substitution combinations were designed and named as TP(i+4) 1&2, TP(i+4) 1&3; TP(i+4) 1&4; TP(i+4) 2&3; TP(i+4) 2&4; TP(i+4) 3&4; TP(i+4) 1&2&3; and TP(i+4) 1&2&4, TP(i+4) 2&3&4. We evaluated the activity and cell selectivity of the peptide synthesized in the first step and selected TP(i+4) 1&2 as the basis of the modification in the second step. Therefore, we carried out a reasonable replacement of the hydrophobic surface. The amino acids where Trp replaces T8 A12, L7 V11, and K6 G10 are named as TP(i+4) 1&2&5, TP(i+4) 1&2&6, and TP(i+4) 1&2&7, respectively. Similarly, their two and three different pairs of combinatorial substitutions were also designed and named as TP(i+4) 1&2&5&6, TP(i+4) 1&2&5&7, TP(i+4) 1&2&6&7, and TP(i+4) 1&2&5&6&7.

Secondary structure determination of designed peptides in 50% solution of 2,2,2-trifluoroethanol (TFE, mainly used to simulate the hydrophobic environment of bacterial cell membranes) and 30 mM sodium dodecyl sulfate (SDS, used to simulate the negatively charged environment of bacterial cell membranes). The cytotoxic and hemolysis properties of the synthetic peptides were investigated, and the antibacterial activity of these peptides was also tested. The stability of the peptide was verified by physiological salt and serum. To further explore the mechanism of peptide action, we used the permeability of outer membrane (OM) and inner membrane to conduct experiments. To directly observe the peptide action mode, we used scanning electron microscopy (SEM) and transmission electron microscopy (TEM) to directly observe the bacterial morphology after peptide treatment. Meanwhile, the potential application of the target peptide *in vivo* was evaluated by mouse peritonitis model.

## Experimental Section

### Bacterial Strains and Materials

The bacteria strains *Escherichia coli* ATCC 25922, *Escherichia coli* UB1005, *Salmonella typhimurium* ATCC 14028, *Salmonella typhimurium* C7731, *Pseudomonas aeruginosa* ATCC 27853, *Pseudomonas aeruginosa* PAO1, *Staphylococcus aureus* ATCC 29213, *Staphylococcus aureus* ATCC 25923, Methicillin-resistant *Staphylococcus aureus* (MRSA) 43300, *Enterococcus faecalis* ATCC 29212, and *Staphylococcus epidermidis* ATCC 12228 were obtained from the College of Veterinary Medicine, Northeast Agricultural University (Harbin, China).

### Synthesis and Structural Analysis of Peptides

All the amphiphiles designed in this study were synthesized by GL Biochem (Shanghai) Ltd. (Shanghai, China) using solid-phase peptide synthesis.

The secondary structure of the peptides (150 μm) was measured at wavelengths ranging from 195 to 250 nm using a J820 spectropolarimeter (Jasco, Tokyo, Japan). The mean residue ellipticity was converted using the following equation:


(1) $$\begin{array}{l} {\text{θ}}_{\text{M}}=\left({\text{θ}}_{\text{obs}}\times 1,000\right)/\left(c\times l\times n\right) \end{array}$$

Where θ_obs_ is the observed ellipticity corrected for the buffer, c is the concentration of the peptides, l is the path length, and n is the number of amino acids in the peptides (Crusca et al., 2018).

### Determination of Antimicrobial Activity

The bacteria were cultured overnight in the MHB medium and transferred to the new MHB the next day until the bacteria reached the exponential growth stage. The bacteria were diluted with MHB to 2 × 10^5^ CFU/ml. About 50 μl of polypeptide and equal volume of bacterial suspension was added to a 96-well plate to give the final concentration of polypeptide between 1 and 128 μm. The last two columns are negative (MHB only) and positive (bacterial and MHB) controls and incubated at 37°C for 18–24 h. Optical density was measured at an absorbance wavelength of 492 nm using a microplate reader (Tecan GENios F129004, Tecan, Austria). At least three independent measurements were taken. Each trial was conducted two times in parallel. A total of three independent assays were carried out.

### Preliminary Toxicity Test

The hemolytic activity of the amphiphiles was assessed by determining the hemolytic rate of the peptide to hRBCs. The cytotoxicity of each peptide was assessed with IPEC-J12 and HEK 293T cells. The specific experimental method is the same as the previous study (Xu et al., 2020; Xue et al., 2021). Each trial was conducted two times in parallel. A total of three independent assays were carried out.

### Salts and Serum Stability Assays

The salt and serum stability of polypeptides were determined by improved MIC method (Lv et al., 2014). The salt and serum stability of polypeptides were determined by improved MIC method. Peptides were incubated with BSA containing different final concentrations of salt ions (150 mM NaCl, 4.5 mM KCl, 6 μm NH_4_Cl, 8 μm ZnCl_2_, 1 mM MgCl_2_, 2 mM CaCl_2_, and 4 μm FeCl_3_) (Wang et al., 2018). To determine the stability of the peptide in serum, peptide was incubated with different proportions of serum (50%, 100%) at 37°C for 4 h. The following steps are the same as those for antimicrobial activity. Each trial was conducted two times in parallel. A total of three independent assays were carried out.

### Drug Resistance Assessment

Continuous culture method was used to evaluate the drug resistance of peptides. Colistin was used as model antibiotic to test the resistance of *E. coli* 25922. First, minimum inhibitory concentration (MIC) was determined for peptide and Colistin Next, the bacteria with each sub-MIC concentration were diluted to 2 × 10^5^ CFU/ml with MHB for the MIC determination of the next passage. The process was repeated 30 times.

### Germicidal Kinetic Test

The bacteria cultured to logarithmic growth phase were diluted to OD600 nm = 0.4 (3 × 10^8^-4 × 10^8^ CFU/ml) and diluted 1,000 times in phosphate buffer saline (PBS). The peptides with 1, 2, 4, 8, and 16 times the minimum inhibitory concentration (MIC) were incubated with the bacterial solution in a polypropylene test tube with a total volume of 1 ml at different time intervals (3, 5, 15, 30, 60, and 120 min). About 50 μl of the mixed solution was absorbed and diluted to an appropriate multiple (100 or 1,000). About 20 μl was extracted and evenly coated on MHA solid medium plate. After overnight incubation at 37°C, the colony number was calculated and the colony number curve was plotted for each time point. A total of three parallels per trial and four trials were repeated.

### *In vivo* Efficacy Assay (Peritonitis–Sepsis Model)

The protocol used in this *in vivo* test was approved by the Animal Care Use Committee of Northeast Agricultural University (NEAU-[2011]-9), and all the animal care and treatment protocols were in line with the standards stipulated in the Regulations on The Management of Experimental Animals of Heilongjiang Province, China (revised in 2016).

A total of 32 female C57BL/6 mice (weighing about 20 g) aged from 6 to 8 weeks were selected. The mice in each group were divided into 4 groups by blind method (*n* = 8): (a) control group (saline), (b) infection group (*E. coli* 25922) (c) treatment group (*E. coli* 25922 + TP(i+4) 1&2&5. (d) treatment group (*E. coli* 25922 + Colistin). Mice in control group were intraperitoneally injected with 100 μl normal saline, and mice in other groups were intraperitoneally injected with 100 μl (~1 × 10^8^ CFU/ml) diluted bacterial solution. The peptide treatment group was intraperitoneally injected with TP(i+4) 1&2&5 (5 mg/kg) at 0 and 1 h after infection, and the control group and antibiotic treatment group were injected with normal saline and colistin (1 mg/kg), respectively. The mice were then euthanized 12 h after infection. After weighing liver, lung, spleen, and kidney, the bacterial load of each organ was determined, and the histopathological changes in the organ sections were analyzed by H&E staining. Serum levels of inflammatory factors IL-6, IL-1β, and TNF-α were detected by ELISA kits (Shanghai Jinma).

### Lipopolysaccharide (LPS) and Lipoteichoic acid (LTA) Neutralization Assays

The LPS and LTA binding affinities of the peptides was assessed with a fluorescent dye BC displacement method. In brief, LPS or LTA was mixed with BC dye at final LPS/LTA and BC concentrations of 50 and 5 μg/ml. The dye was incubated in the dark for 4 h, and the polypeptide was mixed with the dye. Fluorescence was measured using a fluorescence spectrophotometer (Hitachi, Japan) at an excitation wavelength = 580 nm and emission wavelength = 620 nm. LPS-binding affinities were calculated using the following equation:


(2) $$\begin{array}{l} \%\text{Δ}F\left(AU\right)=\left[\left(F_{\text{t}}-F_{\text{n}}\right)/\left(F_{\text{p}}-F_{\text{n}}\right)\right]\;\times 100\% \end{array}$$

Where *F*_t_ is the fluorescence measured after peptide treatment, *F*_n_ is the negative control (the initial fluorescence of BC with a lack of the peptides), and *F*_p_ is the positive control (the fluorescence measured after polymyxin B (10 μg/ml) treatment). Because of its strong binding affinities, polymyxin B was used as the positive control (Kumar and Shin, 2020).

### Outer Membrane (OM) Permeability Tests

The OM permeability of polypeptides was evaluated by fluorescent dye NPN. *E. coli* 25922 in the exponential phase were centrifuged at 5,000 *g* for 5 min at 4°C to discard the MHB, and the remaining bacterial cells were washed three times with HEPES (5 mM, pH = 7.4, containing 5 mM glucose) and resuspended with HEPES to OD600 nm = 0.2. Next, NPN dye (10 μm) and bacterial suspension were incubated for 30 min at 37°C in the dark. Afterward, peptides of equal volume and different concentrations were mixed. Fluorescence leakage induced by polymyxin B (20 μg/ml) was used as positive control. Fluorescence was measured using a fluorescence spectrophotometer (Hitachi, Japan) at an excitation wavelength = 350 nm and emission wavelength = 420 nm. The formula is as follows:


(3) $$\begin{array}{l} \%NPN\;uptake=\left[\left(F_{\text{t}}-F_{\text{n}}\right)/\left(F_{\text{p}}-F_{\text{n}}\right)\right]\;\times 100 \end{array}$$

Where *F*_t_ is the fluorescence measured after peptide treatment, *F*_n_ is the negative control (the initial fluorescence of NPN with a lack of peptides), and *F*_p_ is the positive control (the fluorescence measured after polymyxin B (10 μg/ml) treatment). Owing to its strong OM permeability, polymyxin B was used as the positive control.

### Cytoplasmic Membrane (CM) Depolarization

Fluorescent dye DiSC3(5) method was used to determine the CM depolarization ability of polypeptides. *E. coli* 25922 and *S. aureus* 29213 in the exponential phase were centrifuged at 5,000 *g* for 5 min at 4°C to discard the MHB, and the remaining bacterial cells were washed three times with HEPES (5 mM, pH = 7.4, containing 20 mM glucose) and resuspended with HEPES to OD600 nm = 0.05. Then, DiSC3(5) dye (0.4 μm) and bacterial suspension were incubated for 1 h at 37°C in the dark. Next, 0.1 M KCl was added and then incubated with the bacteria for 30 min, and 2 ml of the mixture was added to each well of a 24-well plate. Afterward, fluorescence was measured using a fluorescence spectrophotometer (Hitachi, Japan) at an excitation wavelength = 622 nm and emission wavelength = 670 nm until a stable reduction in the fluorescence was achieved. Subsequently, the peptides at different concentrations were added to the 24-well plate, and the fluorescence was measured to 1,500 s.

### Flowcytometry Analysis

The damage of peptide to bacterial membrane was evaluated by Becton-Dickinson, USA. The bacteria were cultured to logarithmic stage, washed with PBS, and modulated to OD600 nm = 0.2. The peptides with different concentrations were incubated with bacteria at 37°C for 1 h. Then 1 h later, it was incubated with PI (10 μg/ml) at 4°C for 30 min. Samples without peptide treatment were used as controls.

### Fluorescence Imaging

Fluorescence microscopy was used to further evaluate the ability of peptides to destroy bacterial membranes. The peptides with different concentrations were incubated with bacteria at 37°C for 1 h. Then, SYTO 9 green fluorescent nucleic acid staining and PI red fluorescent nucleic acid staining were performed for 30 min. No peptide-treated bacteria were used as controls. Next, images are obtained using a fluorescence microscope.

### Scanning Electron Microscopy (SEM) and Transmission Electron Microscopy (TEM) Analysis

The bacteria were cultured to logarithmic stage, washed with PBS, and suspended again to OD600 nm = 0.2. About 4 μm of peptide with bacteria was incubated for 60 min, with no peptide as control. The method refers to the previous method in our laboratory (Lai et al., 2019; Yu et al., 2021).

### Statistical Analysis

For statistical analyses, ANOVA was performed using SPSS software, one-way ANOVA was used across multiple groups (one-way ANOVA), and *p* < 0.05 was considered to indicate an extremely significant difference.

## Results and Discussion

### Modification and Structural Analysis of Peptide

**Figure 1:**
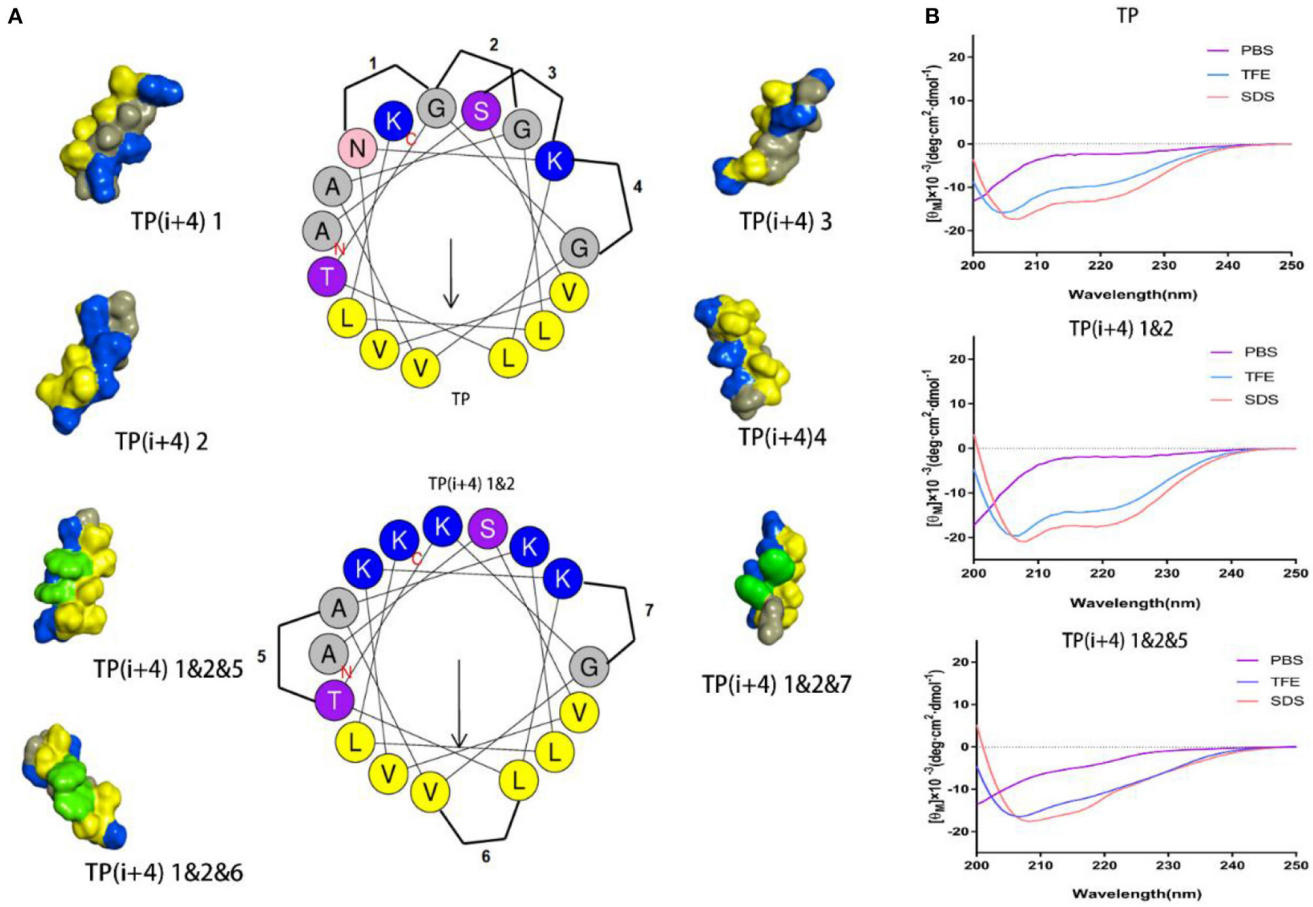
**(A)** Helical wheel projections of the TP and TP(i+4) 1&2, and schematic diagrams of alternate positions. The three-dimensional structure of these peptides was predicted on I-TASSER (Yang and Zhang, 2015), where blue represents positively charged amino acids, yellow represents hydrophobic amino acids, green represents Trp replacement sites, and gray represents other amino acids. **(B)** CD spectra of the TP, TP(i+4) 1&2, and TP(i+4) 1&2&5.

The specific substitution positions and naming are shown in Figure 1, 1–4 are the substitution positions of polar surface positively charged amino acids, and paired Lys substitutions N5 G9, G9 G13, S2 K6, and K6 G10 are named as 1, 2, 3, and 4, respectively. Because it is substituted based on the hydrogen bond formation position (i i+4) on the original peptide TP, so it is uniformly named as TP(i+4). Therefore, the peptides designed by substituting Lys for the N5 G9, G9 G13, S2 K6, and K6 G10 positions of the TP peptide were named as TP(i+4) 1, TP(i+4) 2, TP(i+4) 3, and TP(i+4) 4. Subsequently, considering whether the substitution has a cumulative effect, two and three pairs of different substitution combinations were subsequently designed (TP(i+4) 1&2, TP(i+4) 1&3, TP(i+4) 1&4, TP(i+4) 2&3, TP(i+4) 2&4, TP(i+4) 3&4, TP(i+4) 1&2&3, TP(i+4) 1&2&4, and TP(i+4) 2&3&4). In the second step, the most active TP(i+4) 1&2 was selected as the basis for the second transformation. The amino acids where Trp replaces T8 A12, L7 V11, and K6 G10 are named as TP(i+4) 1&2&5, TP(i+4) 1&2&6, and TP(i+4) 1&2&7. Similarly, their two and three different pairs of combinatorial substitutions were also designed and named as TP(i+4) 1&2&5&6; TP(i+4) 1&2&5&7, TP(i+4) 1&2&6&7, and TP(i+4) 1&2&5&6&7. The key physical and chemical parameters of the designed and synthesized peptides and the projection of the spiral wheel are shown in Table 1 and Supplementary Figure S2.

**Table 1:** Sequence and key physicochemical parameters of amphiphiles.

**Peptide**	**Sequence**	**Measured MW (Da)**	**Theoretical MW(Da)^**a**^**	**Net charge**	**μH^**b**^**	**H^**c**^**
TP	ASVVNKLTGGVAGLLK-NH_2_	1525.83	1526.84	2	0.516	0.379
TP(i+4) 1	ASVV**K**KLT**K**GVAGLLK-NH_2_	1611.02	1612.03	4	0.594	0.325
TP(i+4) 2	ASVVNKLT**K**GVA**K**LLK-NH_2_	1668.07	1669.08	4	0.629	0.315
TP(i+4) 3	A**K**VVN**K**LTGGVAGLLK-NH_2_	1566.92	1567.94	3	0.574	0.379
TP(i+4) 4	ASVVN**K**LTG**K**VAGLLK-NH_2_	1596.95	1597.96	3	0.516	0.377
TP(i+4) 1&2	ASVV**K**KLT**K**GVA**K**LLK-NH_2_	1682.14	1683.15	5	0.646	0.291
TP(i+4) 1&3	A**K**VV**KK**LT**K**GVAGLLK-NH_2_	1655.12	1653.13	5	0.652	0.293
TP(i+4) 1&4	ASVV**KK**LT**KK**VAGLLK-NH_2_	1682.14	1683.15	5	0.591	0.291
TP(i+4) 2&3	A**K**VVN**K**LT**K**GVA**K**LLK-NH_2_	1709.17	1710.19	5	0.688	0.256
TP(i+4) 2&4	ASVVN**K**LT**KK**VA**K**LLK-NH_2_	1739.19	1740.27	5	0.631	0.253
TP(i+4) 3&4	A**K**VVN**K**LTG**K**VAGLLK-NH_2_	1638.05	1639.06	4	0.575	0.318
TP(i+4) 1&2&3	A**K**VV**KK**LT**K**GVA**K**LLK-NH_2_	1723.24	1724.15	6	0.704	0.231
TP(i+4) 1&2&4	ASVV**KK**LT**KK**VA**K**LLK-NH_2_	1753.26	1754.28	6	0.646	0.229
TP(i+4) 2&3&4	A**K**VVN**K**LT**KK**VA**K**LLK-NH_2_	1780.29	1781.30	6	0.690	0.194
TP(i+4) 1&2&5	ASVVKKL**W**KGV**W**KLLK-NH_2_	1882.38	1883.40	5	0.610	0.536
TP(i+4) 1&2&6	ASVVKK**W**TKG**W**AKLLK-NH_2_	1842.27	1843.29	5	0.739	0.389
TP(i+4) 1&2&7	ASVVK**W**LTK**W**VAKLLK-NH_2_	1869.34	1870.35	4	0.676	0.634
TP(i+4) 1&2&5&6	ASVVKK**WW**KG**WW**KLLK-NH_2_	2042.51	2043.53	5	0.693	0.635
TP(i+4) 1&2&5&7	ASVVK**W**L**W**K**W**V**W**KLLK-NH_2_	2069.58	2070.60	4	0.525	0.879
TP(i+4) 1&2&6&7	ASVVK**WW**TK**WW**AKLLK-NH_2_	2029.47	2030.49	4	0.764	0.732
TP(i+4) 1&2&5&6&7	ASVVK**WWW**K**WWW**KLLK-NH_2_	2229.71	2230.73	4	0.617	0.978

a *The theoretical molecular weight (MW) of the peptides was calculated using the Prot Param tool.*

b *Hydrophobic moment (μH).*

c *The mean hydrophobicity (H) was analyzed using HeliQuest (http://heliquest.ipmc.cnrs.fr/cgi-bin/ComputParamsV2.py)*.

The synthesized amphiphiles were designed to measure their molecular weights by mass spectrometry (MS) and reversed-phase high-performance liquid chromatography (RP-HPLC). As shown in Table 1, the measured molecular weight is basically consistent with the theoretical molecular weight.

The secondary structure of these peptides in different solutions was determined by circular dichroism (CD), and the results are shown in Figure 1B and Supplementary Figure S1. CD results show that there is a strong negative peak at 208 nm and a weak negative peak at 222 nm. This indicates that these peptides exhibit α-helical structure in membrane simulation solution (Lee et al., 2013). Meanwhile, we also analyze the α-helical content (Supplementary Figure S3) in different solutions. Lys replacing the hydrogen bond formation position improves the α-helical tendency of the peptide compared to TP. Compared with TP(i+4) 1&2, the α-helical tendency of the derivatives obtained from the hydrophobic surface replaced by Trp decreased to a certain extent. Through the determination of secondary structure, we found that the introduction of Trp reduced the helix content in different amplitude. The interaction between amino acid side groups has a great influence on the formation of helix structure (Louis-Jeune et al., 2012).

### Antimicrobial Activity

The MICs of the designed peptides against gram-negative bacteria and gram-positive bacteria are shown in Table 2. In order to investigate the effect of substitution of positive charge at ith and i+4th positions on the antibacterial activity, 14 peptides were designed. Based on the experimental research, we found that compared with the original peptide TP, all of the peptides except TP(i+4) 3 showed inhibitory activity against negative bacteria. The TP(i+4) 1&2 of the first and second pairs replaced at the same time had the best activity. Based on the TP(i+4) 1&2, the effect of Trp replacing the hydrophobic active center and expanding the hydrophobic surface at both ends on the activity of the peptide was investigated. Compared with the TP(i+4) 1&2, other peptides showed compromised antimicrobial activity against both gram-negative and gram-positive bacteria. At the same time, TP(i+4) 1&2&5 was screened out by analysis and comparison.

**Table 2:** The MICs^a^ (μm) of the peptides.

**Peptide**	**Gram-negative bacteria**						**Gram-positive bacteria**				
	***E. coli*** **25922**	***E. coli*** **UB1005**	***S. typhimurium*** **14028**	***S. typhimurium*** ***7731***	***P. aeruginosa*** ***27853***	***P. aeruginosa*** ***PAO1***	***S. aureus*** **29213**	***S. aureus*** **25923**	***S. aureus*** **43300**	***E. faecalis*** **29212**	***S. epidermidis*** **12228**
TP	>128	>128	>128	>128	>128	>128	>128	>128	>128	>128	>128
TP(i+4) 1	16	16	128	64	64	64	>128	>128	>128	>128	>128
TP(i+4) 2	16	8	128	64	64	32	>128	>128	>128	>128	>128
TP(i+4) 3	>128	>128	>128	>128	>128	>128	>128	>128	>128	>128	>128
TP(i+4) 4	16	16	128	64	64	64	>128	>128	>128	>128	>128
TP(i+4) 1&2	4	4	32	16	16	8	>128	>128	>128	>128	>128
TP(i+4) 1&3	64	16	>128	>128	128	64	>128	>128	>128	>128	>128
TP(i+4) 1&4	8	8	64	32	32	16	>128	>128	>128	>128	>128
TP(i+4) 2&3	32	8	128	128	128	128	>128	>128	>128	>128	>128
TP(i+4) 2&4	8	8	64	16	32	8	>128	>128	>128	>128	>128
TP(i+4) 3&4	128	64	>128	>128	>128	128	>128	>128	>128	>128	>128
TP(i+4) 1&2&3	32	8	128	64	64	16	>128	>128	>128	>128	>128
TP(i+4) 1&2&4	4	4	64	32	32	8	>128	>128	>128	>128	>128
TP(i+4) 2&3&4	16	16	64	64	128	8	>128	>128	>128	>128	>128
TP(i+4) 1&2&5	1	2	2	4	2	2	2	2	2	2	2
TP(i+4) 1&2&6	4	8	16	32	32	8	64	128	64	128	64
TP(i+4) 1&2&7	4	4	4	8	4	4	2	4	4	4	4
TP(i+4) 1&2&5&6	1	2	2	4	4	4	8	16	8	8	8
TP(i+4) 1&2&5&7	16	16	32	>128	>128	>128	8	16	16	8	8
TP(i+4) 1&2&6&7	4	4	4	8	8	4	2	2	4	2	2
TP(i+4) 1&2&5&6&7	1	2	2	4	4	4	2	4	4	2	2

a *Minimum inhibitory concentration (MIC, μm) is defined as the minimum concentration of peptide for inhibiting bacterial growth*.

The amount of charge has a direct effect on the activity of the amphiphiles, but with the development of research, the factors that affect the activity are not limited to the amount of positive charge carried by the peptide, but also depend on the degree of charge aggregation. As shown in Figure 1A, we simulated the structural diagrams of these amphiphiles. The substitution of different sites results in distinct charge aggregation of peptides. We know that from our preliminary activity results, the four peptides that replace a pair of amino acids at the ith and i+4th positions on the polarity plane with Lys are in the order of activity, which was TP(i+4) 2 > TP(i+4) 1 ≈ TP(i+4) 4 > TP(i+4) 3. As expected, TP(i+4) 2-positive charges closely distributed on one side had better activity and TP(i+4)1 and TP(i+4)4 with similar degree of charge aggregation and as we predicted, they had similar antibacterial activity. Compared with the other three peptides, TP(i+4) 3 has a lower charge density. At the same time, we also found through the analysis of its secondary structure TP(i+4) 3 which has a lower helical content than the TP. Many previous reports have suggested that the tendency to form α-helical structures is correlated with the antibacterial activity of α-helical AMPs (Zhao et al., 2013). According to the helical content data in Supplementary Figure S3, the contents of TP(i+4) 3 in TFE and SDS are 54.85 and 61.94%. Thus, we conclude that secondary structure is another important reason for its low activity. Based on the investigation of the initial activity of these amphiphiles substituted by Lys, we find that the stack effect of replacing two pairs is better than a pair effect and the three pairs effect. The antibacterial activity did not increase as the replacement logarithm increased. Excessive positive charge residue is easy to produce electrostatic obstruction which is not conducive to the activity of the peptide. Among them, the relationship between charge and biological activity is not linear and seems to show a threshold. The results showed that the antimicrobial activity of the peptides did not increase when the positive charge was above the threshold. The peptide in this study had the best antibacterial activity at a net charge of +5. This is consistent with the previous research (Lyu et al., 2019). TP(i+4) 1&2 with the best activity was selected to explore the effects of Trp replacement at the H-H positions on the hydrophobic center and the sides of the hydrophobic surface on the peptide activity and cell selectivity. We found that the design and synthesis of TP(i+4) 1&2&6 by replacing the position of the hydrophobic center did not increase the activity as expected, but decreased the activity compared with TP(i+4) 1&2. We discussed this result and concluded that Trp as a common amino acid in natural AMPs, which carries a large indole side chain, so the interaction between the side chains is more obvious (Lopes et al., 2013; Feng et al., 2020). As shown in Figure 1A, the Trp residues in the hydrophobic center were in contact with each other and wound together. Due to the interaction of side chains, the ability of the tryptophan side chain to insert into the bacterial plasma membrane was weakened, so the hydrophobic surface of TP(i+4) 1&2&6 was reduced as a whole, affecting its activity. In the experimental process, we also found that the H-H position of Trp at both ends of the hydrophobic surface significantly improved the activity of the amphiphiles, and the TP(i+4) 1&2&5 showed best compromised antimicrobial activity against both gram-negative and gram-positive bacteria. However, TP(i+4) 1&2&5&7 was significantly lower than TP(i+4) 1&2&6&7 and TP(i+4) 1&2&5&6 by analyzing the activity of multiple pairs of substituted peptides. By analyzing its hydrophobicity, we found that the value of TP(i+4) 1&2&5&7 was 0.879, much higher than that of TP(i+4) 1&2&6&7 and TP(i+4) 1&2&5&6. We infer that the relationship between hydrophobicity and bioactivity is not linear. When the hydrophobicity exceeded the threshold, the antibacterial activity of the antimicrobial peptides decreased rather than increase.

### Hemolysis and Cytotoxicity of Peptides

**Figure 2:**
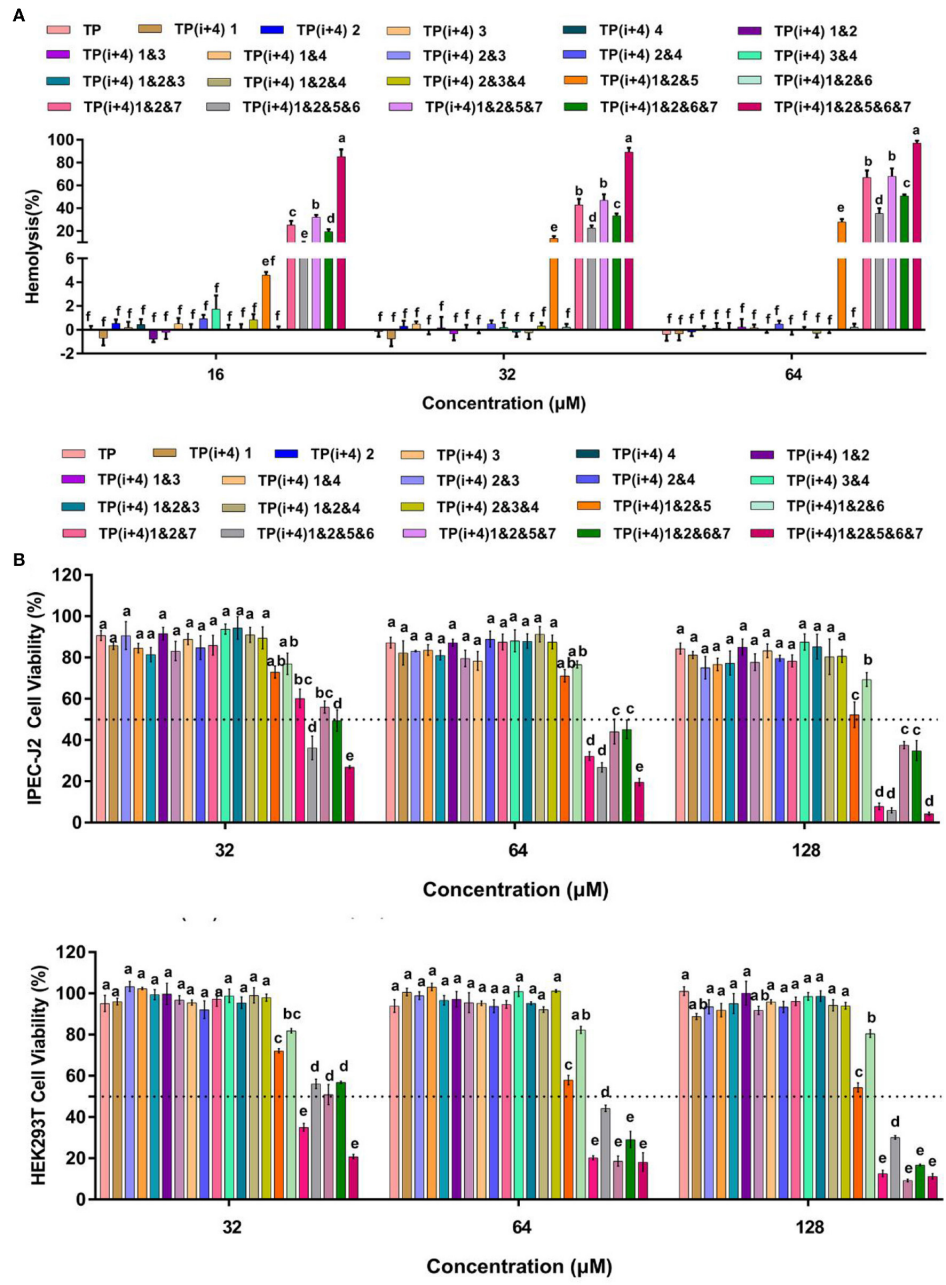
**(A)** is the hemolytic activity of the designed peptides, **(B)** is the toxicity of the peptides designed for IPEC-J2 and HEK293T cells. A total of three independent tests were conducted for each test, and the data were expressed as mean ± SEM. Differences between groups at the same concentration were analyzed by one-way ANOVA. The values with different superscripts (a, b, c, d, e, f) indicate a significant difference (*p* < 0.05).

The increasing number of experiments has prompted us to continue to investigate the obstacles to the practical application of these amphiphiles. In addition to their antimicrobial activity, the toxicity of these peptides in practical use is another important consideration (Zhu et al., 2020). To understand the effect of different substitution sites on the cytotoxicity of the peptides, we measured the cytotoxicity and hemolysis activity of these designed peptides. It was observed that Lys substituted peptides had weak cytotoxic effects on HEK 293T and IPEC-J2 cells (Figure 2B and Supplementary Figure S4). Similarly, these peptides rarely cause hemolysis even at maximum concentrations of 64 μm (Figure 2A). Through the significance analysis of these peptides, it was found that there was no significant difference between these 14 peptides. However, toxicity measurements of the amphiphiles based on the substitution of Trp on the hydrophobic surface showed that the introduction of hydrophobic amino acids enhanced the toxicity of the peptides. Except for TP(i+4) 1&2&6 and TP(i+4) 1&2&5, the other peptides showed high cytotoxicity and hemolysis. TP(i+4) 1&2&6 and TP(i+4) 1&2&5 maintained ~50% cell viability even at 128 μm. TP(i+4) 1&2&5 showed lower hemolysis than other peptides, and the hemolysis rate was <50% below 64 μm.

By comparing the toxicity of polypeptides formed by Lys substitution, it was found that paired Lys substitution at H-H formation sites increased activity without increasing toxicity. Trp is a hydrophobic amino acid with aromatic side chains. It is a special amino acid in peptide design. Previous studies have pointed out that introducing amino acids will also bring toxicity while improving activity. Therefore, at the beginning of the design, we predicted that selecting Trp as hydrophobic amino acid to substitute H-H position would bring certain toxicity to the peptide. Thus, as expected, the introduction of Trp increases the activity and toxicity of these peptides. However, by analyzing the toxicity of the peptides, we unexpectedly found that the substitution of tryptophan for TP(i+4) 1&2&6 in the hydrophobic center did not increase its toxicity. Meanwhile, the virulence of TP(i+4) 1&2&5&6 and TP(i+4) 1&2&6&7 was lower than that of TP(i+4) 1&2&5&7. We infer that the location of hydrophobic centers can effectively reduce peptide toxicity by Trp substitution. For further evaluating the cell selectivity of the peptides, we also calculated the SI which defined as the ratio of MHC to the geometric mean (GM) (Dufourc, 2008). As shown in Table 3, compared with other peptides TP(i+4) 1&2&5 has a good therapeutic index (SI = 64), indicating that TP(i+4) 1&2&5 has a greater application potential in the body.

**Table 3:** The MHC, GM, and TI values of the peptides.

**Peptide**	**MHC(μM)^**a**^**	**Geometric mean (GM**, **μM)**^**b**^			**Therapeutic index (TI)** ^**c**^		
		**Gram-negative bacteria**	**Gram-positive bacteria**	**All**	**Gram-negative bacteria**	**Gram-positive bacteria**	**All**
TP	>128	128.00	128.00	128.00	1.00	1.00	1.00
TP (i+4) 1	>128	45.25	128.00	76.11	2.83	1.00	1.68
TP (i+4) 2	>128	35.92	128.00	67.81	3.56	1.00	1.89
TP (i+4) 3	>128	128.00	128.00	128.00	1.00	1.00	1.00
TP (i+4) 4	>128	45.25	128.00	76.11	2.83	1.00	1.68
TP (i+4) 1&2	>128	10.08	128.00	35.92	12.70	1.00	3.56
TP (i+4) 1&3	>128	71.84	128.00	95.89	1.78	1.00	1.33
TP (i+4) 1&4	>128	20.16	128.00	50.80	6.53	1.00	2.56
TP (i+4) 2&3	>128	64.00	128.00	90.51	2.00	1.00	1.41
TP (i+4) 2&4	>128	16.00	128.00	45.25	8.00	1.00	2.83
TP (i+4) 3&4	>128	114.04	128.00	120.82	1.12	1.00	1.06
TP (i+4) 1&2&3	>128	35.92	128.00	67.81	3.56	1.00	1.89
TP (i+4) 1&2&4	>128	14.25	128.00	42.71	8.90	1.00	2.98
TP (i+4) 2&3&4	>128	32.00	128.00	64.00	4.00	1.00	2.00
TP (i+4) 1&2&5	>128	2.00	2.00	2.00	64.00	64.00	64.00
TP (i+4) 1&2&6	>128	12.7	85.45	32.94	10.08	1.50	3.89
TP (i+4) 1&2&7	64.00	4.49	3.48	3.95	14.25	18.39	16.19
TP (i+4) 1&2&5&6	64.00	2.52	9.19	4.81	25.40	6.96	13.30
TP (i+4) 1&2&5&7	64.00	50.80	10.56	23.16	1.26	6.06	2.76
TP (i+4) 1&2&6&7	64.00	5.04	2.30	3.40	12.70	27.83	18.80
TP (i+4) 1&2&5&6&7	8.00	2.52	2.64	2.58	3.17	3.03	3.10

a *MHC is the concentration of peptide that causes hemolysis of 50% of human red blood cells. When 50% hemolysis was not observed at 128 μm, 128 μm was used to calculate the therapeutic index.*

b *The geometric mean (GM) of MIC value (all strains in Table 2). When no antibacterial activity was detected at 128 μm, 128 μm was used to calculate the therapeutic index.*

c *The selectivity index (SI) was calculated as MHC/GM. Lager values indicate higher therapeutic potential (Ong et al., 2014)*.

### Salts and Serum Stability

The complex physiological environment is an important reason to limit the application of AMP *in vivo* (Shao et al., 2020). According to the above analysis results, TP(i+4) 1&2 and TP(i+4) 1&2&5 were selected for stability study. The stability difference between the selected optimal peptide for positive charge replacement and the optimal peptide for hydrophobic replacement was discussed. Therefore, we used an improved MIC method to evaluate the effects of different salt ion environments on peptide activity. Table 4 shows that different salt ion environments have no or only partial influence on MIC values of TP(i+4) 1&2&5. However, for peptide TP(i+4) 1&2, salt ions have a great influence on its activity. As indicated in Table 4, the stability of TP(i+4) 1&2&5 and TP(i+4) 1&2 in 50 and 100% serum was also determined. TP(i+4) 1&2&5 active serum had less effect compared to TP(i+4) 1&2.

**Table 4:** MIC (μm) values of designed peptides in the presence of physiological salts and serum.

**Peptide**	**Control**	**Physiological salts**							**GM**	**Serum**		**GM**
		**Na^**+**^**	**K^**+**^**	**Mg^**2+**^**	**Zn^**2+**^**	** ${\text{NH}}_{4}^{+}$ **	**Fe^**3+**^**	**Ca^**2+**^**		**50%**	**100%**	
***E. coil*** **25922**												
TP (i+4) 1&2	4	>64	8	32	2	8	8	>64	39.75	16	32	22.62
TP (i+4) 1&2&5	1	4	2	2	1	2	1	4	2.22	2	2	2.00
***S. aureus*** **29213**												
TP (i+4) 1&2	>64	>64	>64	>64	>64	>64	>64	>64	>64	>64	>64	>64
TP (i+4) 1&2&5	2	2	1	2	1	2	2	2	1.75	4	8	5.65

Although the peptides exhibit good antibacterial activity, its antibacterial activity will be reduced or even lost under physiological conditions of salt and serum. Poor stability is one of the main factors that limit the clinical application potential of AMPs. In many studies on natural peptides, it is not difficult to find that cations such as Na^+^ and Mg^2+^ are more likely to affect the activity of AMPs (Yu et al., 2011; Chu et al., 2013). Since the electrostatic force between the negative charge on the outer membrane of bacteria and the cationic AMPs attracts each other, therefore, that any factor that reduces the electrostatic interaction between the two, such as high ionic strength, will affect the antibacterial activity of AMPs (Han et al., 2016). However, in this study, TP(i+4) 1&2&5 was shown to have a strong inhibitory effect on gram-negative and gram-positive bacteria in the presence of salt. The TP(i+4) 1&2 of the positive charge replacement design is greatly affected by salt ions. They have the same positive charge and are close to amphiphilicity. However, TP(i+4) 1&2&5 was formed by replacing ith and i+4th with Trp, expanded the hydrophobic surface, and had higher hydrophobicity. It can be inferred that the strategic addition of hydrophobic residues to peptide sequences enhances the affinity of AMPs to bacterial membranes and thus the ability to overcome salt sensitivity. The serum environment had different degrees of inhibition on TP(i+4) 1&2&5 and TP(i+4) 1&2. This phenomenon can be explained using a balance between the free peptide molecule and the protein binding peptide molecule. As more free molecules combine their microbial targets, balance will be gradually released to more peptide molecules, which facilitates maintaining their antibacterial activity (Deslouches et al., 2005). In addition, the antibacterial activity decreased with the increase of serum concentration. This may be due to high concentrations of albumin binding to antimicrobial peptide molecules, which makes the peptide less active (Svenson et al., 2007).

### Antimicrobial Mechanism Research

We evaluated the toxicity and activity of these peptides. Finally, TP, TP(i+4) 1&2, and TP(i+4) 1&2&5 were selected to study the mechanism of action. To make the mechanism description more reasonable and credible, the peptides are implemented in a comprehensive membrane operating system for typical bacteria. The membrane manipulation of *E. coli* 25922 and *S. aureus* 29213 from outside to inside was shown in the main body, from qualitative to quantitative, and from molecular components to morphology. The membrane permeation of gram-negative bacteria and gram-positive bacteria was studied with fluorescent probes, and the direct interaction between peptides and bacteria was observed under various microscopes.

#### LPS and LTA Neutralization Assays

**Figure 3:**
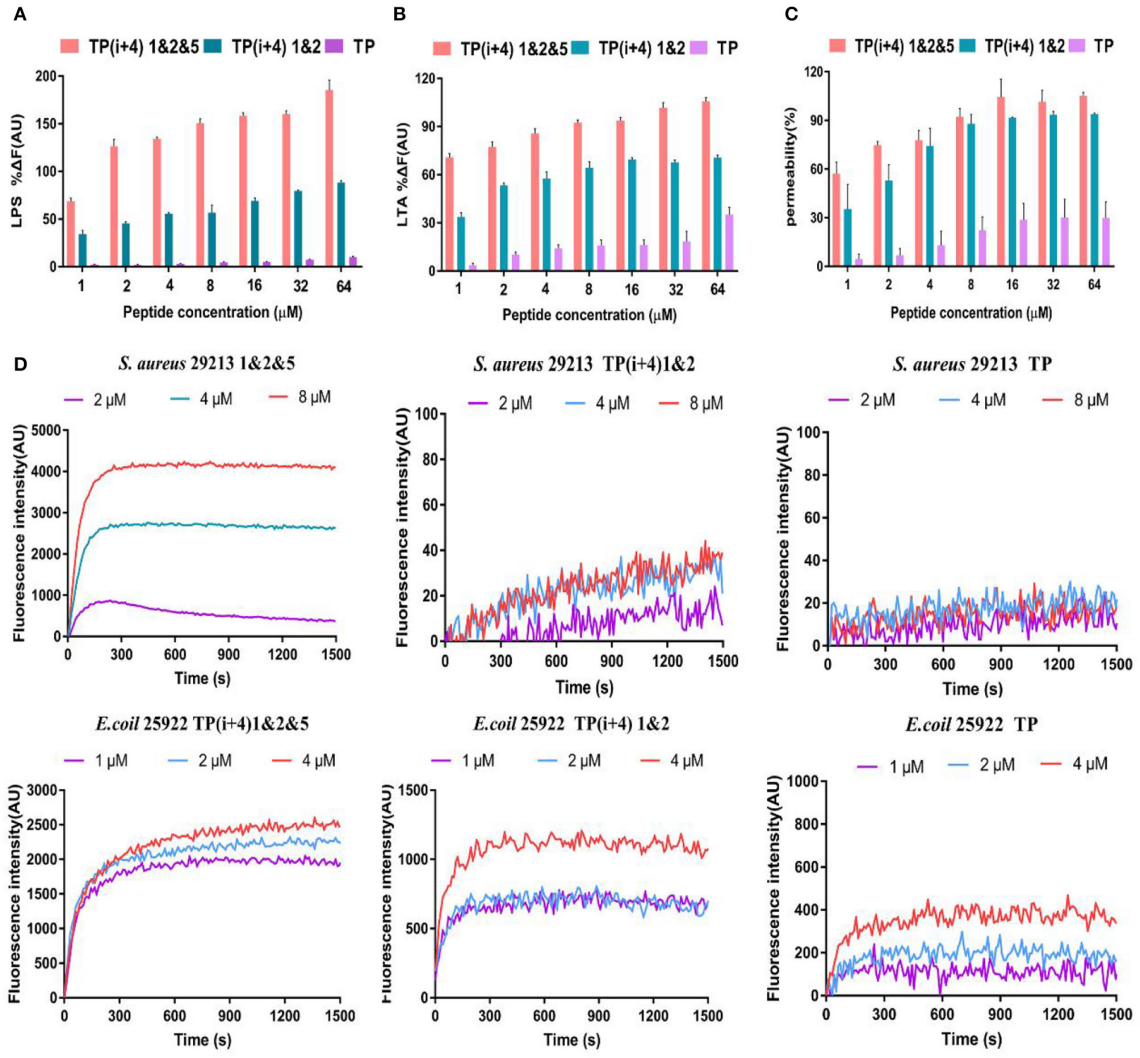
The mechanism of action of peptides, **(A)** and **(B)** are polypeptides to bind binding capabilities on specific substances and LTA on the bacterial membrane. **(C)** OM permeability of the polypeptide. **(D)** Depolarize of the CM of the synthesis of the peptides.

The LPS and LTA are specific components with negative charge on the membrane surface of gram-negative and gram-positive bacteria, respectively, and are the main binding sites of most AMPs through electrostatic attraction (Koller and Lohner, 2014). As shown in Figures 3A,B, the binding affinity of TP(i+4) 1&2 and TP(i+4) 1&2&5 to LPS and LTA increased in a dose-dependent manner, suggesting that the peptides could bind negatively charged components of the membranes of gram-negative and gram-positive bacteria by electrostatic attraction. However, by comparison, it can be seen that the binding ability of TP(i+4) 1&2 to LPS/LTA is not as good as that of TP(i+4) 1&2&5. Many studies have shown that the binding ability of peptides to LPS and LTA is not only limited to positive charge, but also related to the amphiphilic structure of their helices (Wei et al., 2013; Rajasekaran et al., 2019). This explains that TP(i+4) 1&2 and TP(i+4) 1&2&5 with the same charge differ greatly in LPS-binding ability at the same concentration. We infer that the introduction of marginal side Trp reduces the amphiphilic structure of TP(i+4) 1&2&5 and makes TP(i+4) 1&2&5 with the same charge, which have higher LPS-binding ability.

#### Outer Membrane (OM) Permeability

Membrane permeability is one of the most important characteristics of the peptide–membrane interaction, which determines the activity of cationic AMPs (Epand et al., 2010; Koller and Lohner, 2014; Paterson et al., 2017). OM is a special ingredient on the gram-negative fungus film. The permeability of the polypeptide to the OM was explorated with *N*-phenyl-1-naphthylamine (NPN) in the experiment. In general, the hydrophobic probe NPN is excluded by the bacterial outer membrane, but when the bacterial outer membrane is damaged or the structure loosens, the NPN will enter the cell membrane, resulting in the increase of fluorescence intensity. As shown in Figure 3C, here, we demonstrate that synthetic peptides penetrate the outer membrane barrier in a concentration-dependent manner, and the TP(i+4) 1&2&5 were able to penetrate the cell outer membrane adequately, with the percentage of permeabilization of the outer membrane reaching over 100% at peptide concentrations >16 μm. However, the ability of TP to act on bacterial outer membranes is poor.

#### Cytoplasmic Membrane Depolarization of Synthetic Peptides

In addition, we investigated the CM depolarization ability of these synthetic peptides using membrane potential-dependent probe DISC3-5. When cell membranes are permeated and destroyed, DISC3-5 was released into the medium resulting in an increase in fluorescence in the solution. As shown in Figure 3D, in *E. coli* 25922, the degree of TP(i+4) 1&2&5 depolarization was enhanced with increasing polypeptide concentration. However, at low concentrations (1 and 2 μm) of TP(i+4) 1&2, the difference between the curves was not significant since the effective inhibitory concentration was not reached. Overall, TP(i+4) 1&2&5 depolarized the bacterial inner membrane more efficiently. In *S. aureus* 29213, since TP(i+4) 1&2 and TP were inactive against positive bacteria, they did not appear to be able to effectively depolarize the bacterial inner membrane in this assay.

### Resistance Development

**Figure 4:**
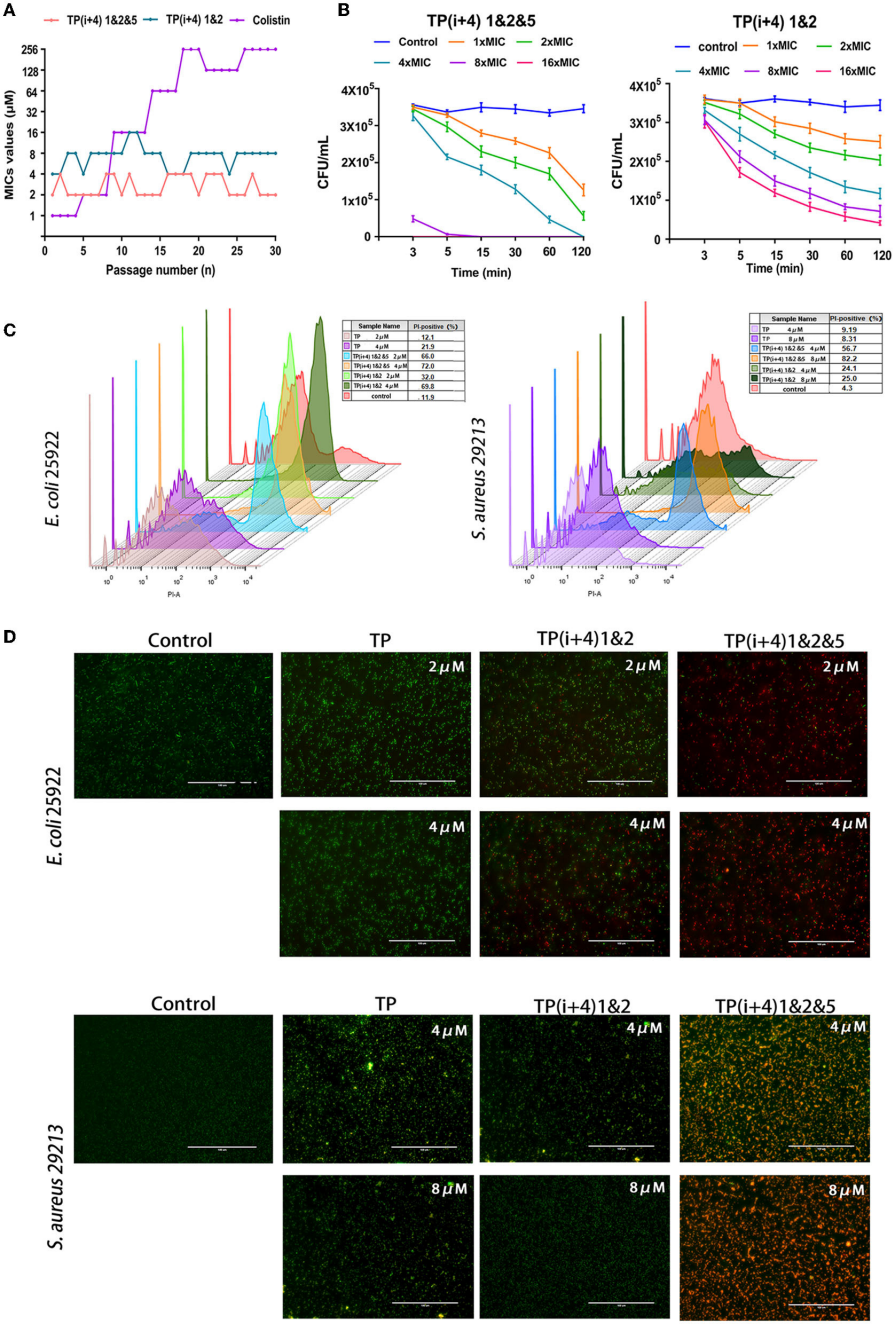
**(A)** Resistance developments in the presence of a sub-MIC concentration of peptides TP(i+4) 1&2, TP(i+4) 1&2&5, and colistin. **(B)** Bactericidal kinetics of TP(i+4) 1&2&5 and TP(i+4) 1&2 against *E. coli* 25922; **(C)** FCM was used to analyze the damage of peptide to bacterial membrane. **(D)** Fluorescence microscopy, green signal: SYTO 9, red signal: PI.

The likelihood of antimicrobial peptides causing bacterial resistance is considered low but bacterial resistance is still an important research parameter (Andersson et al., 2016; Marston et al., 2016; Cw et al., 2021). Figure 4A shows the potential of TP(i+4) 1&2, TP(i+4) 1&2&5, and colistin to induce bacterial resistance. Colistin resistance appeared as early as the sixth generation, with a 256-fold increase in MIC values after 30 generations and the antibacterial efficacy of TP(i+4) 1&2&5 and TP(i+4) 1&2 did not change during the trial. The results of the mechanism of action study in the previous step showed that the designed peptides exert their antibacterial action mainly by penetrating and disrupting bacterial cell membranes. Bacteria can only resist the physical disruption of the peptide by altering their membrane structure, and therefore, the possibility of AMPs inducing resistance in bacteria is low. Polymyxin, as a control, showed a 256-fold increase in MIC values after 30 generations of polymyxin at the end of the assay period. It has been shown that resistance to polymyxin is associated with plasmid-mediated delivery of the gene MCR1 between strains and the addition of cationic moieties (L-Ara4N and pEtN) to the LPS. In summary, TP(i+4) 1&2&5 and TP(i+4) 1&2 act in a similar manner to most AMPs, where the positive charge on the surface of AMPs is electrostatically attracted to the negative charge of the bacterial membrane, and when the concentration of AMPs reaches a certain threshold, their hydrophobic ends insert into the bacterial membrane disrupting it and causing it to leak until death. This mode of action greatly reduces the likelihood of drug resistance in microorganisms and gives AMPs an unprecedented advantage in preventing the development of bacterial drug resistance.

### Bactericidal Kinetics

Thus, the resistance of *E. coil* 25922 to TP(i+4) 1&2 and TP(i+4) 1&2&5 and the bactericidal dynamic curve of the target amphiphilic peptide against the infected strain were evaluated before preclinical testing. The time killing curve of *E. coli* 25922 was determined, and it was found that the target peptide caused the decrease of bacteria within 0–15 min, and then, the bactericidal rate tended to slow down. However, neither peptide at 1 × MIC concentration could provide sufficient power to completely kill the pathogen within 2 h (Figure 4B). With the increase of peptide concentration, TP(i+4) 1&2&5 at 4 × MIC can completely kill *E. coli* 25922 within 2 h. 8 × MIC and 16 × MIC can kill pathogens within 3 min. TP(i+4) 1&2 do not completely kill *E. coli* 25,922 even at 16 × MIC concentration. The hydrophobicity is an important factor that affects the antibacterial efficiency. TP(i+4) 1&2 has poor bactericidal effect because TP(i+4) 1&2 and TP have low hydrophobicity.

### Flow Cytometry (FCM) and Fluorescence Microscope

Using FCM and the nuclear fluorescent dye PI, which can penetrate the damaged membrane, the damaging activity of peptide on the bacterial membrane was evaluated. Meanwhile, fluorescence microscopy was used to observe the ability of the peptide to destroy bacterial membranes (Shi et al., 2006). The degree of membrane rupture (PI fluorescence signal) increased with the increase of peptide concentration (Figure 4C). TP(i+4) 1&2&5 caused more than 80% of *E coli* 25922 rupture and more than 70% of *S aureus* 29213 rupture. Compared with TP(i+4) 1&2 and TP, TP(i+4) 1&2&5 has stronger ability of sterilization by membrane breaking. At the same time, fluorescence imaging analysis was performed using the non-membrane permeable nucleic acid dye propidium iodide (PI) and the membrane permeable nucleic acid dye SYTO 9. The fluorescence images showed a similar trend to the FCM results (Figure 4D). These results further confirm that TP(i+4) 1&2 and TP(i+4) 1&2&5 kill bacteria by destroying the integrality of the bacterial membranes.

### SEM and TEM

**Figure 5:**
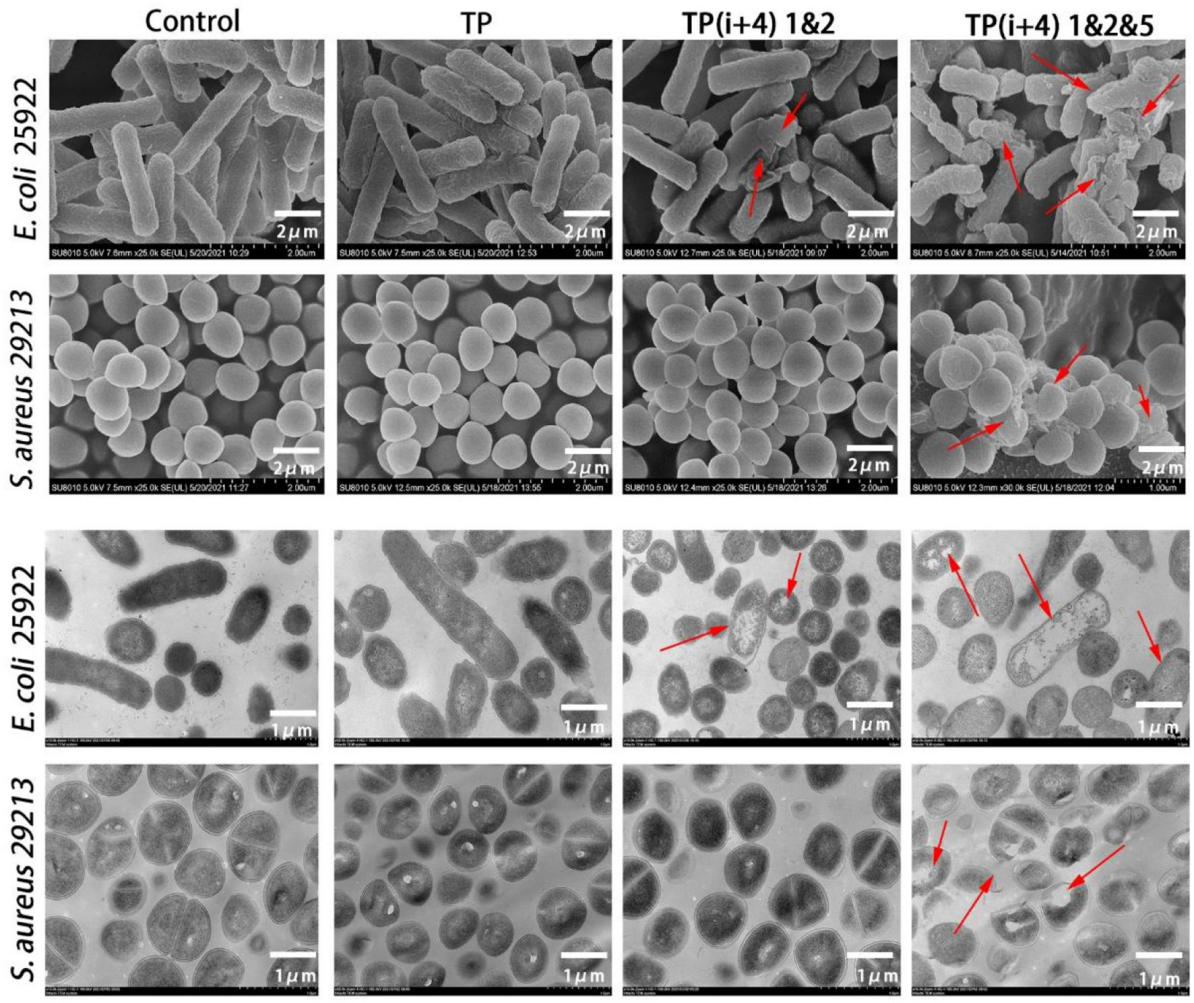
SEM and TEM micrographs of *E. coli* 25922 and *S. aureus* 29213 treated with TP, TP(i+4) 1&2 and TP(i+4) 1&2&5 for 60 min. SEM: the scale bar is 2 μm. TEM: the scale bar is 1 μm. The red arrows point to areas of obvious damage to the bacterial film.

For a more intuitive observation of the activity mechanism of the peptide, SEM and TEM further examined the effects of TP, TP(i+4) 1&2, and TP(i+4) 1&2&5 on the cell morphology of *E. coli* 25922 and *S. aureus* 29213 (Figure 5). The control group was *E. coli* 25,922 and *S. aureus* 29,213, which were not treated with peptide. The test results could be observed that their bacterial cell membrane surface was full and smooth with no signs of rupture and the bacterial membrane was in good condition. The TP-treated bacterial cell membranes showed slight wrinkles on the surface, but no obvious pores or fragmentation was observed. The TP(i+4) 1&2- and TP(i+4) 1&2&5-treated *E. coli* 25922 showed visible folds and large pores and breaks on the surface of the bacterial cell membrane, but TP(i+4) 1&2 was less effective than TP(i+4) 1&2&5. Since there is no activity against TP(i+4) 1&2-positive bacteria, the cell membrane state of *S. aureus* 29213 treated with TP(i+4) 1&2 in the figure is not significantly different from the control. The effects of TP, TP(i+4) 1&2, and TP(i+4) 1&2&5 on the membrane ultrastructure of *E. coli* 25922 and *S. aureus* 29213 were further visualized using TEM. It was clearly observed that the membranes of the control group without peptide treatment were clear and intact, with no structural changes and dense internal cell structure. The outer and plasma membranes of *E. coli* 25922 and *S. aureus* 29213 treated with TP(i+4) 1&2&5 were clearly separated, the cell membranes were ruptured, and the cytoplasm was leaking. However, since TP(i+4) 1&2 had no activity against the positive bacteria, there was no significant difference between the treated *S. aureus* 29213 and the control.

### *In vivo* Efficacy

**Figure 6:**
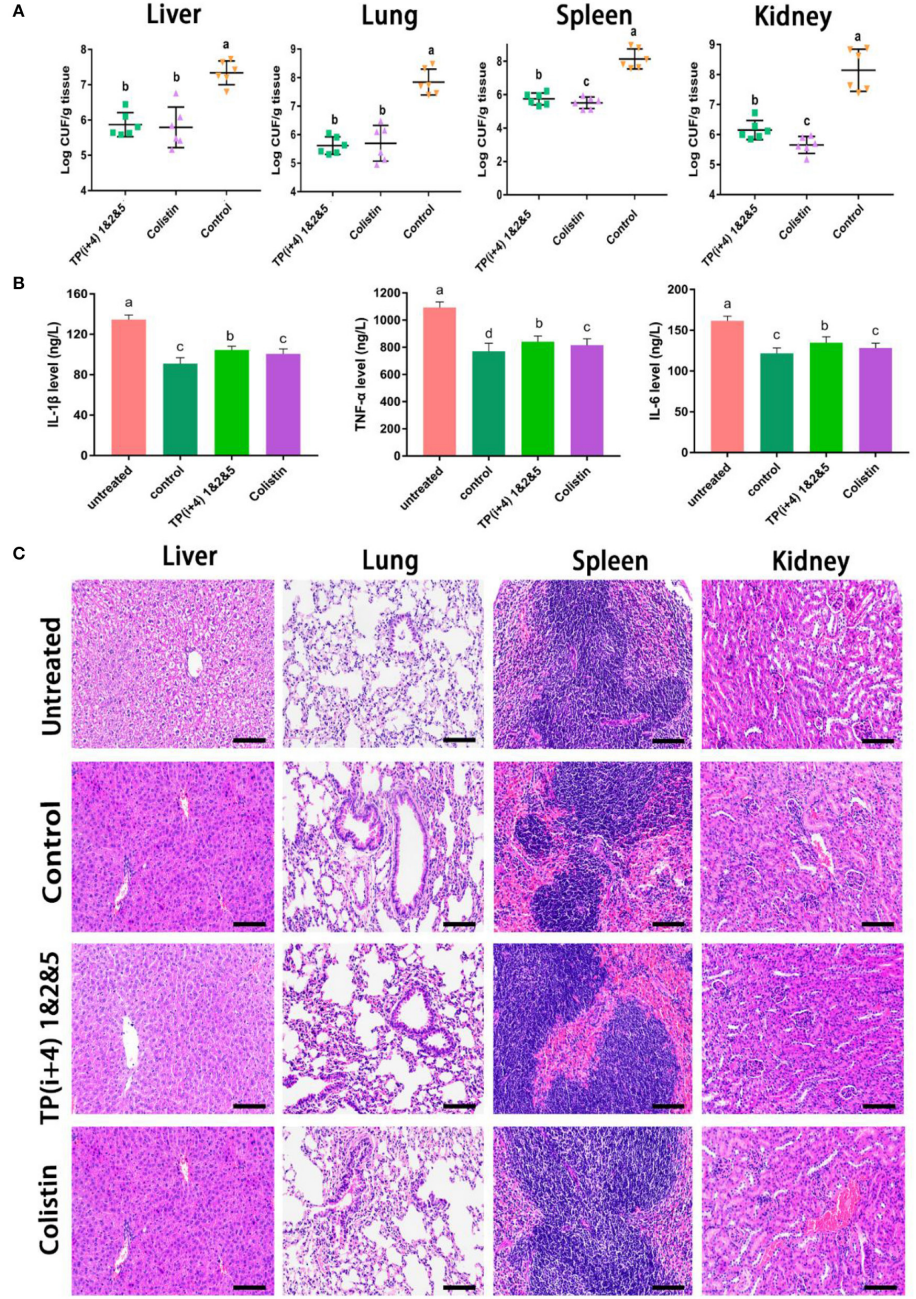
*In vivo* therapeutic evaluation of the target peptide TP(i+4) 1&2&5 in a mouse model of bacterial peritonitis **(A)** Bacterial load in different organs (*n* = 6) **(B)** Regulation of peptides on serum cytokine (IL-6, IL-1β, and TNF-α) levels **(C)** Peritonitis C57BL/6 mice liver, spleen, kidney, lung tissue pathological morphology observation. Scale bar: 100 μm. **(A,B)** Values with different superscripts (a, b, c, d) indicate a significant difference (*p* < 0.05).

Preliminary *in vitro* antibacterial activity and toxicity determination and analysis provided the basis for AMPs *in vivo*. Through the comprehensive study and screening of the activity and toxicity of these synthetic peptides as well as the mechanism of action, the potential application of the ultimate target amphiphiles *in vivo* was studied. We evaluated the *in vivo* therapeutic effects of TP(i+4) 1&2&5 in the *E. coli* 25922-mediated C57BL/6 mouse peritonitis model and selected colistin as a control. TP(i+4) 1&2&5 and colistin treatment significantly reduced bacterial load in multiple organs (Figure 6A). Histopathological examination showed that TP(i+4) 1&2&5 therapy significantly reduced multi-organ inflammatory response in varying degrees compared with the infected group. The infiltration of lung and hepatitis cells decreased, and the bleeding of kidney and spleen decreased. No significant lesions were observed in these organs after the peptide treatment (Figure 6C). In addition, to evaluate the therapeutic effect of the target peptide *in vivo*, serum levels of interleukin-1β (IL-1β), interleukin-6 (IL-6) and tumor necrosis factor-α (TNF-α) were measured. The test results showed a significant reduction in serum levels of IL-1β, IL-6, and TNF-α compared to the *E. coli* 25922-infected group. IL-1β is considered to be a pro-inflammatory cytokine that plays an important role in the host's defense response to acute infection (Schmidt and Lenz, 2012; Li et al., 2017). IL-6 is a major mediator of sepsis and can cause septic shock through hemorrhage and severe oedema. TNF-α is secreted by immune cells (activated macrophages, monocytes, and T cells). The overexpression of TNF-α is associated with body damage such as apoptosis and tissue inflammation and also induces the recruitment of other inflammatory cells (Jiang et al., 2016; Gao et al., 2021). As the results shown in Figure 6B, the levels of inflammatory factors were significantly lower in the peptide-treated group than in the infected group. The reduction in these proinflammatory cytokines suggests that TP(i+4) 1&2&5 also has potential anti-inflammatory effects *in vivo*.

## Conclusion

In this study, the natural perfect amphiphilic antimicrobial peptide TP was used as a template for modification, and the influence of the systematic substitution of Lys at the hydrogen bond formation position (i, i+4) on the structure and activity of the peptide was investigated. Based on the first step, the influence of the substitution of Trp for the hydrophobic center and the positions i and i+4 on both sides of the peptide activity and cell selectivity was discussed. Although the peptides designed and synthesized varied in size and helices, TP(i+4) 1&2, which had a higher charge density, was found to be more active than other peptides. At the same time, we found that the specific substitution of ith and i+4th positions on both sides of the hydrophobic surface gave better activity. The target peptide TP(i+4) 1&2&5 was selected by comprehensive evaluation that has good activity against gram-negative bacteria and gram-positive bacteria. The present results also show that it still has good activity in the presence of physiological salt and serum and has the ability to penetrate and destroy membrane integrity. At the same time, it has good therapeutic effect on peritonitis model in mice. Together, these results can be generalized to a useful method for designing and optimizing spiral AMPs with enhanced cell selectivity.

## Data Availability Statement

The original contributions presented in the study are included in the article/Supplementary Material, further inquiries can be directed to the corresponding authors.

## Ethics Statement

The animal study was reviewed and approved by Animal Care Use Committee of Northeast Agricultural University (NEAU- [2011]-9).

## Author Contributions

GL and AS designed and conceived this work. GL, XY, HC, BL, CS, YZ, and ZL conducted the main experiments assay. GL wrote the main manuscript text. AS supervised the work and revised the final version of the manuscript. All of the authors have read and approved the final version of the manuscript.

## Funding

The work was supported by the Natural Science Foundation of Heilongjiang Province (TD2019C001) and National Natural Science Foundation of China (32030101, 31872368).

## Conflict of Interest

The authors declare that the research was conducted in the absence of any commercial or financial relationships that could be construed as a potential conflict of interest.

## Publisher's Note

All claims expressed in this article are solely those of the authors and do not necessarily represent those of their affiliated organizations, or those of the publisher, the editors and the reviewers. Any product that may be evaluated in this article, or claim that may be made by its manufacturer, is not guaranteed or endorsed by the publisher.
