# Supplementary material for: Optimization of Antibacterial Activity in Tibetan Swine α-Helix Peptide TP by Site-Directed Mutagenesis
Source: Front Microbiol. 2022 Jul 4;13:864374. doi: 10.3389/fmicb.2022.864374 (PMC9289672; doi:10.3389/fmicb.2022.864374)
Supplement: Supplementary file 1 [file Data_Sheet_1.docx]

**Optimization of Antibacterial Activity in Tibetan Swine α-Helix Peptide TP by Site-directed Mutagenesis**

*Guoyu Li, Xiaojie Yuan, Bowen Li, Hongyu Chen Changxuan Shao Yongjie Zhu, Zhenheng Lai and Anshan Shan**^*^*

*** Corresponding author.

E-mail addresses: asshan@neau.edu.cn (A. Shan)

Laboratory of Molecular Nutrition and Immunity. The Institute of Animal Nutrition, Northeast Agricultural University, Harbin, 150030, P. R. China.

**Key words:** TP; Antimicrobial peptides; Site-directed Mutagenesis; Antibacterial activity;


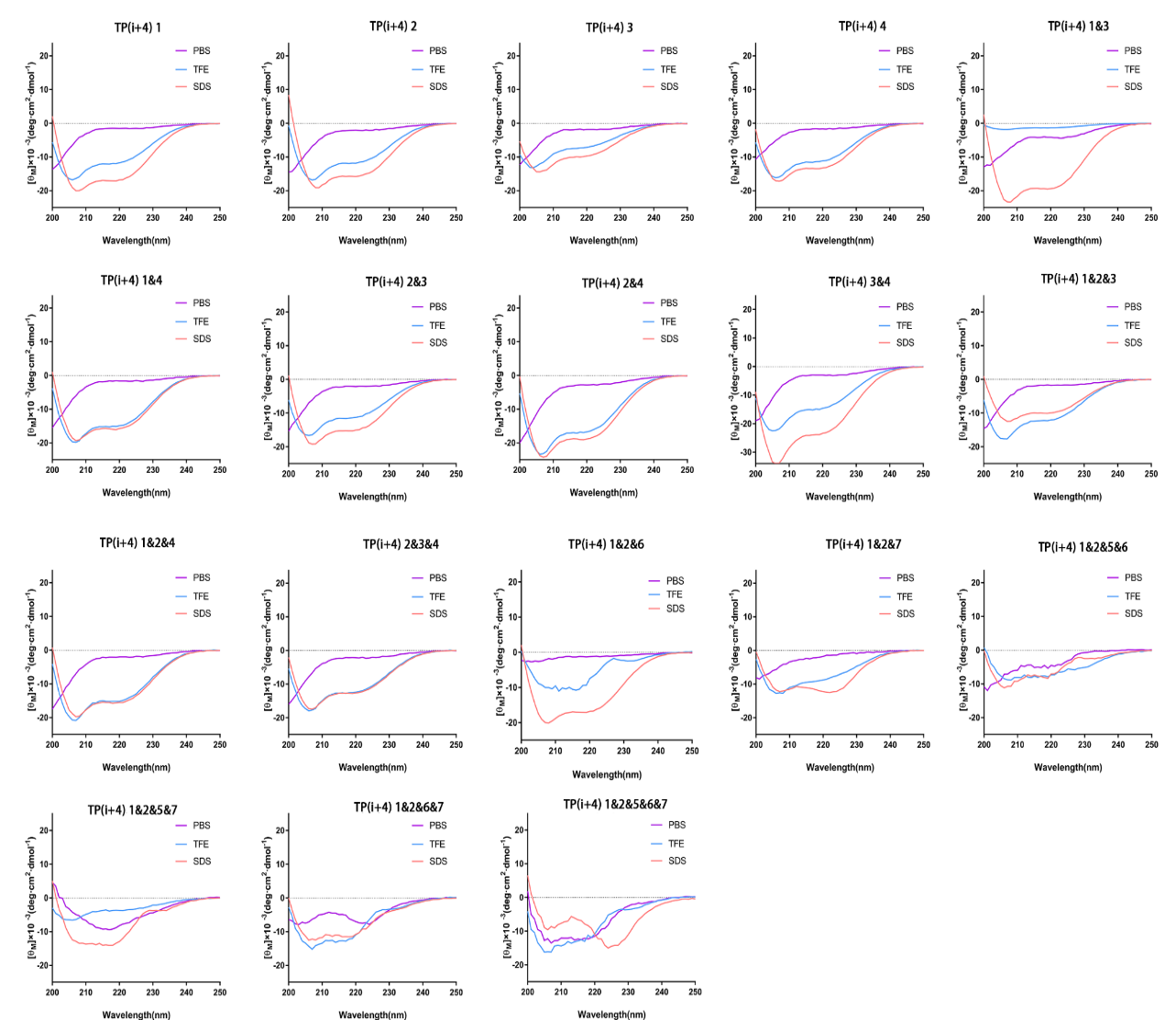
**Figure S1.** CD spectra of the peptides. The peptides were dissolved in 10 mM sodium phosphate buffer,50% TFE and 30 mM SDS.


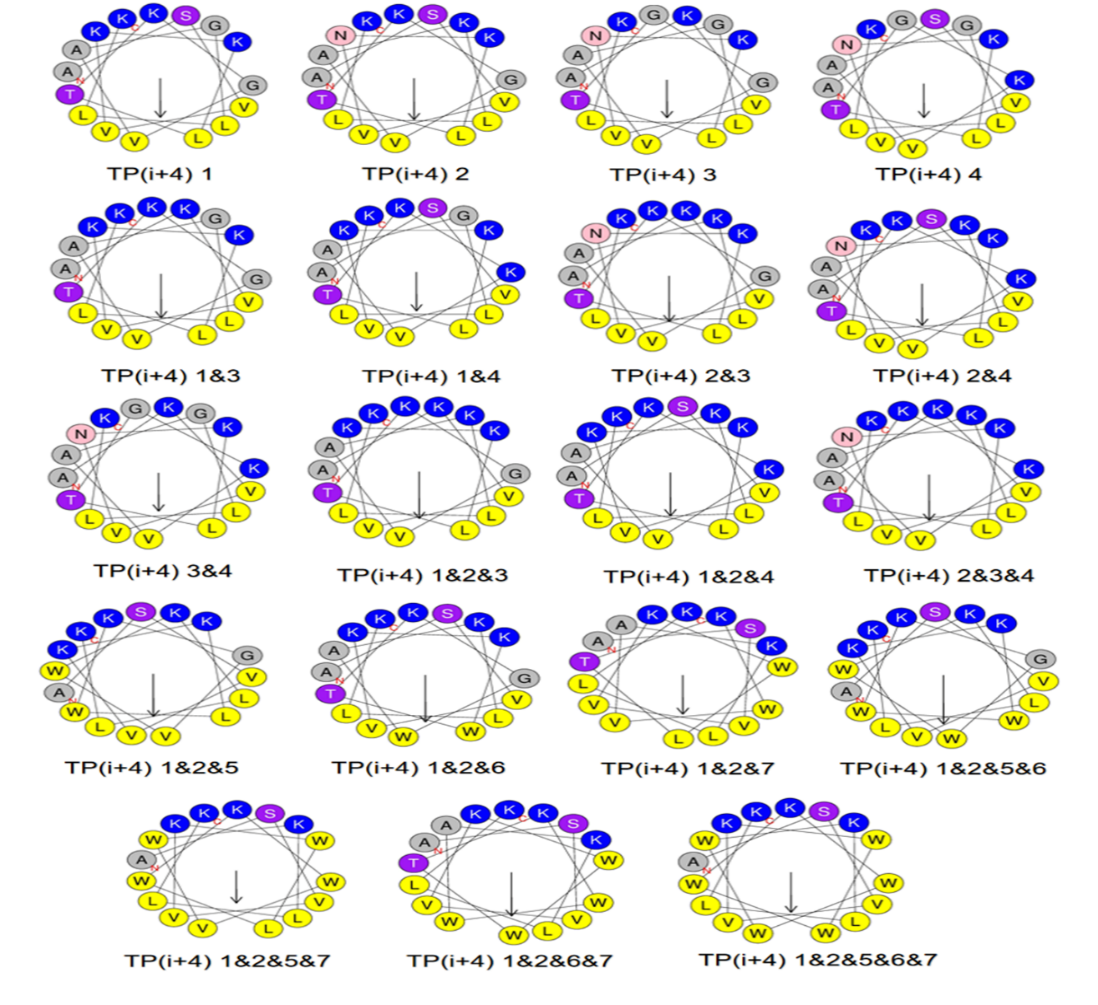


**Figure S2.** Helical wheel projections of the peptides. The output of positively charged residues is blue and that of hydrophobic residues is yellow. The output of hydrophilic residues is purple.


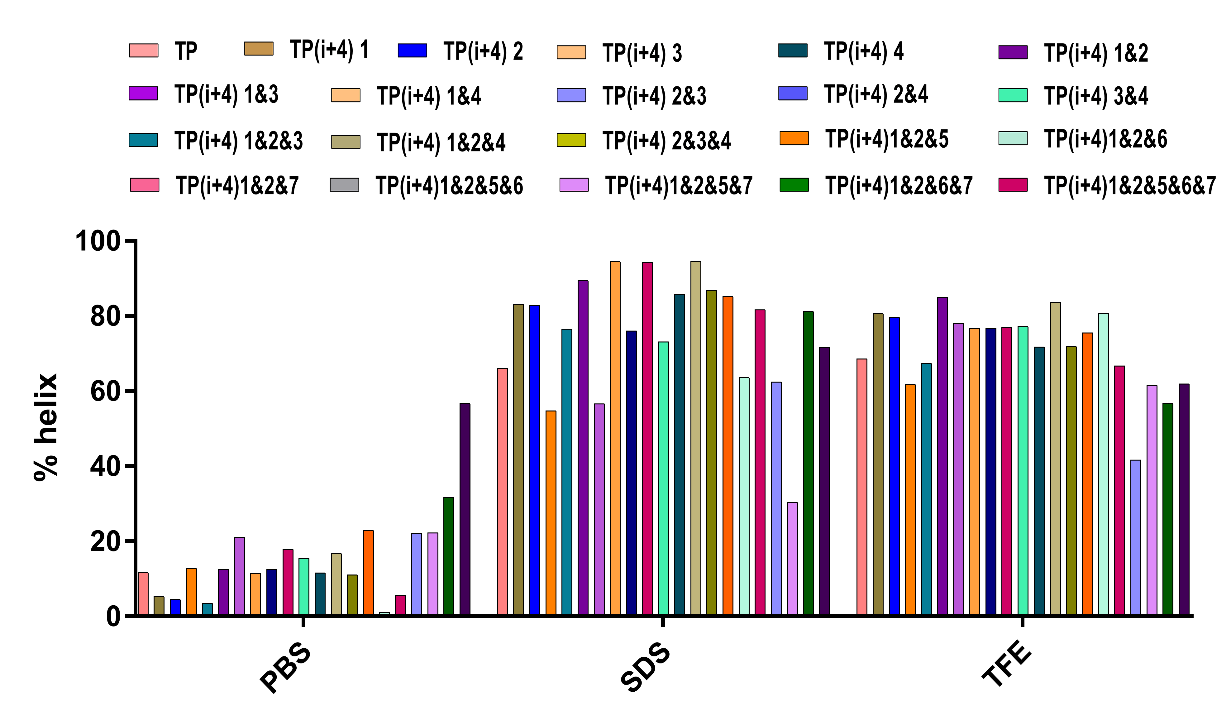


**FigureS3.** Prediction of α-helix content in different solutions by K2D3(http://cbdm-01.zdv.uni-mainz.de/~andrade/k2d3//)


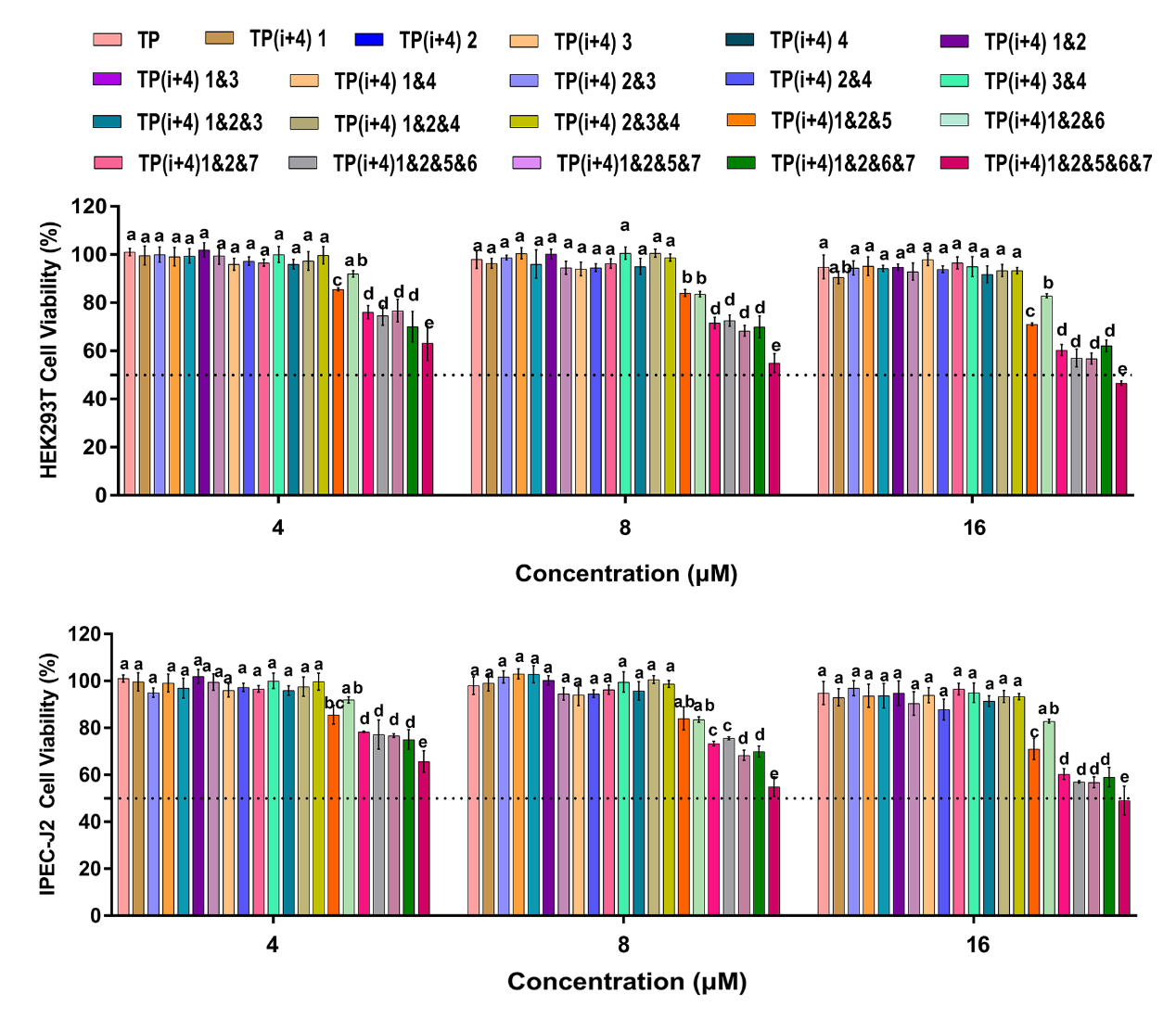


**FigureS4**. Cytotoxicity of the peptides against IPEC-J2 cells and HEK 293T Cells. Each test is performed in 6 replicates on 3 independent occasions, the data are expressed as the mean ± SEM. Differences between groups exposed to the same concentration are determined by one-way ANOVA followed by Tukey’s post hoc analysis (n = 6). The values with different superscripts (a, b, c, d, e) indicate a significant difference (*P* < 0.05).

**FigureS5**. HPLC report of peptides


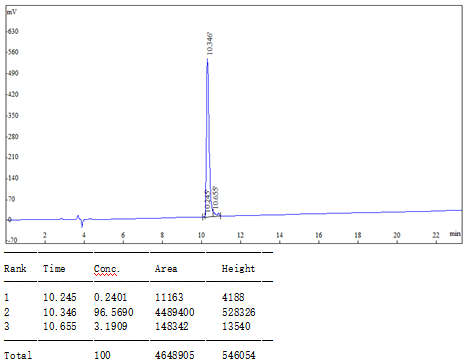


TP


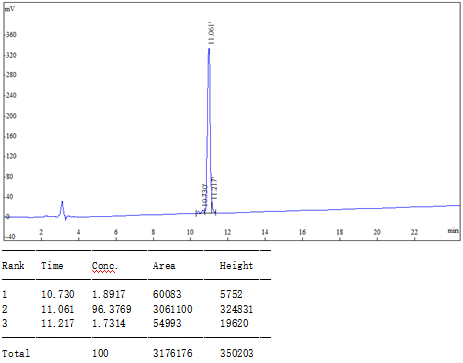


TP(i+4) 1


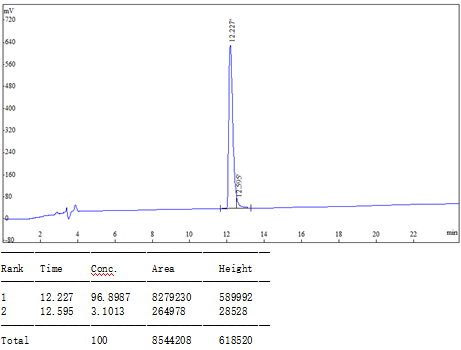


TP(i+4) 2


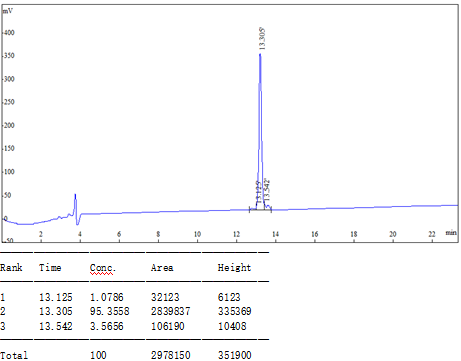


TP(i+4) 3


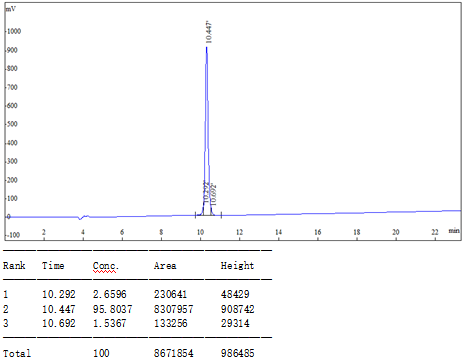


TP(i+4) 4


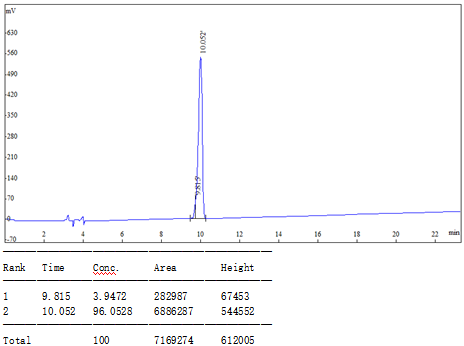


TP(i+4) 1&2


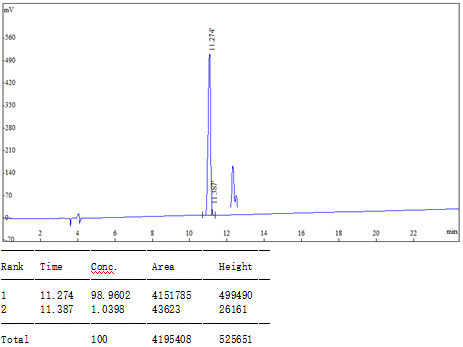


TP(i+4) 1&3


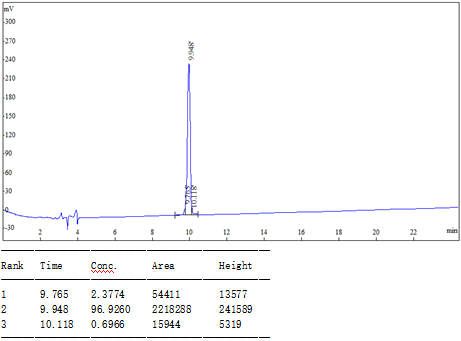


TP(i+4) 1&4


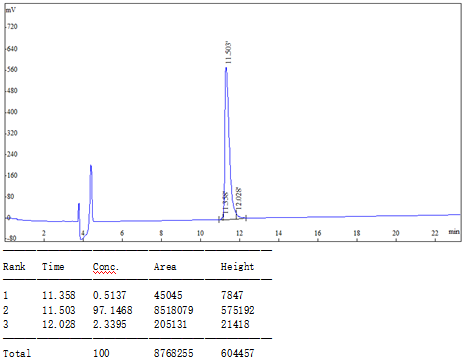


TP(i+4) 2&3


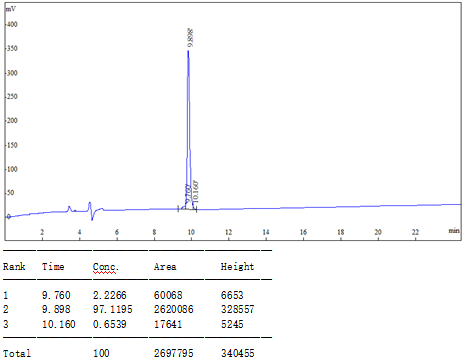


TP(i+4) 2&4


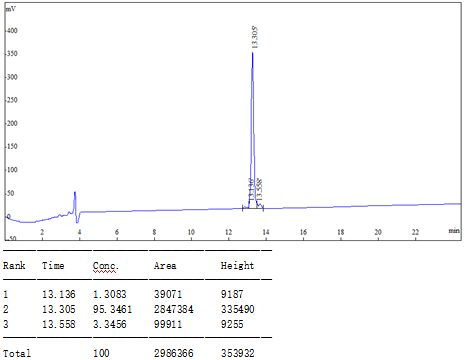


TP(i+4) 3&4


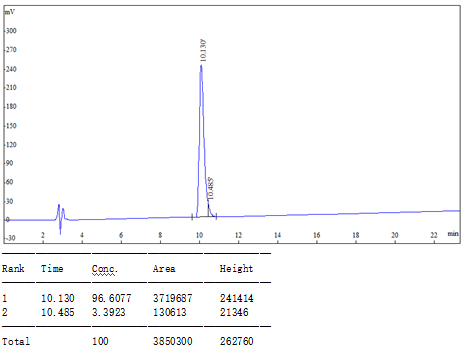


TP(i+4) 1&2&3


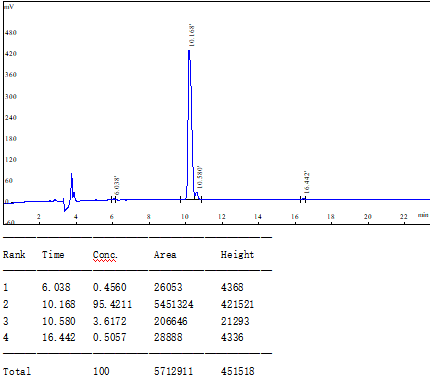


TP(i+4) 1&2&4

**
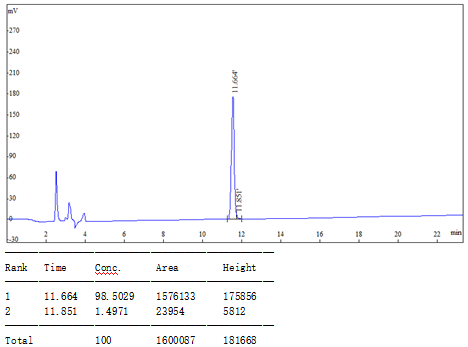
**

TP(i+4) 2&3&4


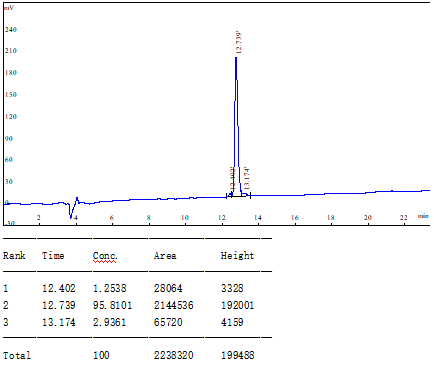


TP(i+4) 1&2&5


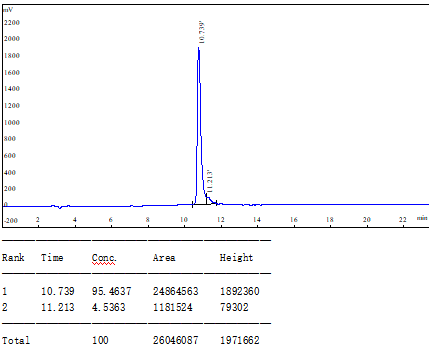


TP(i+4) 1&2&6


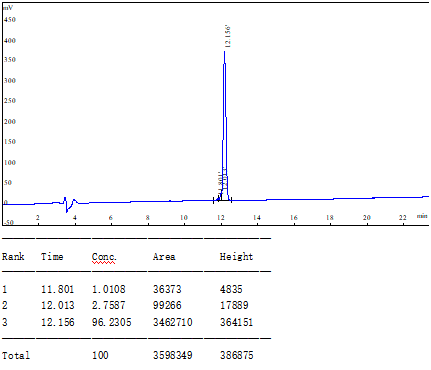


TP(i+4) 1&2&7


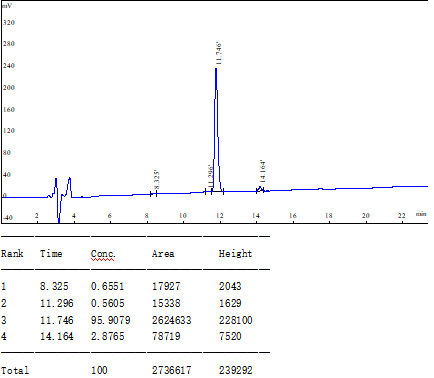


TP(i+4) 1&2&6&7


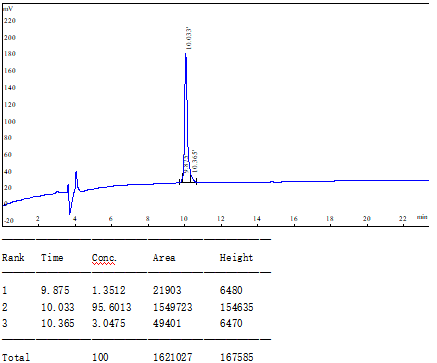


TP(i+4) 1&2&5&6


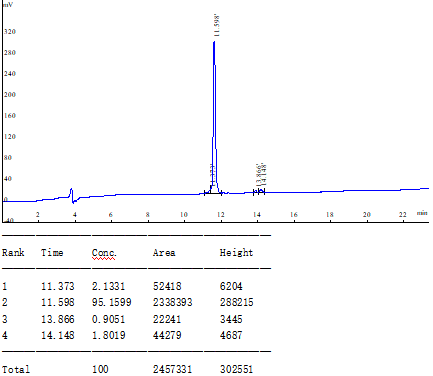


TP(i+4) 1&2&5&7


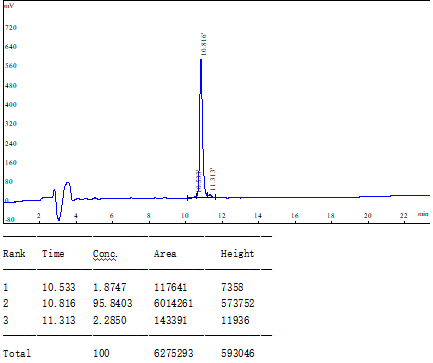


TP(i+4) 1&2&5&6&7
